# Endoscopic resection is a suitable initial treatment strategy for oxyntic gland adenoma or gastric adenocarcinoma of the fundic gland type

**DOI:** 10.1038/s41598-021-86893-w

**Published:** 2021-04-01

**Authors:** Masaya Iwamuro, Chiaki Kusumoto, Masahiro Nakagawa, Sayo Kobayashi, Masao Yoshioka, Tomoki Inaba, Tatsuya Toyokawa, Shinichiro Hori, Shouichi Tanaka, Kazuhiro Matsueda, Takehiro Tanaka, Hiroyuki Okada

**Affiliations:** 1 Department of Gastroenterology and Hepatology, Okayama University Graduate School of Medicine, Dentistry and Pharmaceutical Sciences, 2-5-1 Shikata-cho, Kita-ku, Okayama, Okayama 700-8558 Japan; 2 Department of Gastroenterology, Nippon Kokan Fukuyama Hospital, 1844 Tsunoshita, Daimon-cho, Fukuyama, Hiroshima 721-0927 Japan; 3 Department of Internal Medicine, Hiroshima City Hospital, 7-33 Motomachi, Naka-ku, Hiroshima, 730-8518 Japan; 4 Department of Internal Medicine, Fukuyama City Hospital, 5-23-1 Zao-cho, Fukuyama, Hiroshima 721-8511 Japan; 5 Department of Internal Medicine, Okayama Saiseikai General Hospital, 2-25 Kokutai-cho, Kita-ku, Okayama, Okayama 700-8511 Japan; 6 Department of Gastroenterology, Kagawa Prefectural Central Hospital, 1-2-1 Asahi-cho, Takamatsu, Kagawa 760‑8557 Japan; 7 Department of Gastroenterology, Fukuyama Medical Center, 4-14-17 Okinogami-cho, Fukuyama, Hiroshima 720-8520 Japan; 8 Department of Endoscopy, National Hospital Organization Shikoku Cancer Center, 160 Kou, Minamiumemoto-cho, Matsuyama, Ehime 791-0280 Japan; 9 Department of Gastroenterology, Iwakuni Clinical Center, 1-1-1 Atago-cho, Iwakuni, Yamaguchi 740-8510 Japan; 10 Department of Gastroenterology and Hepatology, Kurashiki Central Hospital, 1-1-1 Miwa, Kurashiki, Okayama 710-8602 Japan; 11 Department of Pathology, Okayama University Graduate School of Medicine, Dentistry, and Pharmaceutical Sciences, 2-5-1 Shikata-cho, Kita-ku, Okayama, Okayama 700-8558 Japan

**Keywords:** Cancer, Cancer therapy, Gastrointestinal cancer

## Abstract

The aim of this study was to reveal the histological features of oxyntic gland adenomas and gastric adenocarcinoma of the fundic-gland type (GA-FG). We retrospectively examined the histological features of 126 lesions of oxyntic gland adenoma and/or GA-FG in 116 patients. The prevalence of oxyntic gland adenomas and GA-FG was approximately equal. The majority of the lesions were resected by endoscopic mucosal resection using a diathermic snare (EMR, n = 42) or endoscopic submucosal dissection (ESD, n = 72). Histologically, there were no lesions with invasion at the level of the muscularis propria or deeper, and lymphovascular invasion was present in 1.6%. Of the ESD and EMR specimens, there were no lesions that were positive for vertical margins. Among the eight GA-FG patients with deep (≥ 500 μm) submucosal invasion, six were treated with endoscopic resection alone, and no recurrence was documented. No patients died of the disease during the median follow-up period of 14.5 months. In conclusion, all lesions were confined to the mucosa or submucosa and were negative for vertical margins. Lymphovascular invasion was present in only 1.6% of the patients. Thus, we believe that endoscopic resection is a suitable initial treatment method for oxyntic gland adenoma and GA-FG.

## Introduction

Gastric adenocarcinoma of the fundic gland type (GA-FG) is an epithelial neoplasm composed of columnar cells with differentiation to chief cells, parietal cells, or both. Since GA-FG was first reported as a single case report in 2007^1^, histological, clinical, endoscopic, and genetic features have been investigated and documented in multiple case reports and case series^2–21^. With the increasing popularity of the disease concept, GA-FG has been formally cited as a variant of gastric adenocarcinoma in the latest version of the classification of gastric neoplasms issued by the World Health Organization (WHO)^22^. Historically, this disease entity was variably named gastric adenocarcinoma with chief cell differentiation^1,5^, gastric neoplasia of the fundic gland (chief cell-predominant) type^2,7,9^, gastric fundic gland-associated neoplasms/polyps^13^, gastric adenocarcinoma of the fundic gland (chief cell predominant type)^14,16^, and chief-cell predominant gastric polyps^15^. According to the WHO classification, a neoplasm confined to the mucosa is called an oxyntic gland adenoma, while a neoplasm with submucosal invasion is classified as GA-FG^22^. Due to their infrequency, treatment strategies for oxyntic gland adenoma and GA-FG have not yet been established. In this study, we included 126 lesions of oxyntic gland adenoma and/or GA-FG from 116 patients across 10 institutions and retrospectively examined the histological features, which is an essential step in the determination of an appropriate initial treatment strategy. The clinical and endoscopic features of this disease have also been summarized.

## Methods

Letters of inquiry were sent to nine collaborating institutions with patients with histologically diagnosed oxyntic gland adenoma and GA-FG, from the Department of Gastroenterology and Hepatology, Okayama University Graduate School of Medicine, Dentistry, and Pharmaceutical Sciences. Histological diagnoses were made based on endoscopic biopsy, endoscopic mucosal resection, endoscopic submucosal dissection, and/or surgical resection. The diagnoses of oxyntic gland adenoma and GA-FG were made based on the presence of intramucosal proliferation of differentiated columnar cells with pale basophilic cytoplasm and mild nuclear atypia, which have been found to mimic the oxyntic (fundic) gland^22^. The diagnoses were supported by positive immunohistochemical staining for pepsinogen I and MUC6 in some patients. We identified 116 patients who were diagnosed with oxyntic gland adenoma and/or GA-FG between October 2008 and February 2020. These patients were retrospectively enrolled in the study.

We retrospectively examined the patients’ sex, age at diagnosis, *Helicobacter pylori* infection status, endoscopic features, histological features, treatments, and prognoses. In patients with two or more lesions, a second or third primary lesion identified within 12 months of the detection of the first lesion were defined as “synchronous” multifocal lesions, while those identified more than 12 months after the detection of the first lesion were classified as “metachronous” multifocal lesions. *H. pylori* infection status was examined by urea breath tests, rapid urease tests, microscopic observations or culture tests on endoscopically biopsied specimens, stool antigen tests, serum or urine antibody tests, or a combination of these methods. Based on these test results, *H. pylori* infection status was classified into one of three groups: (i) uninfected (the gastric mucosa has never been infected by *H. pylori*), (ii) active gastritis (the gastric mucosa is currently infected by *H. pylori*), and (iii) inactive-gastritis (the gastric mucosa was previously infected by *H. pylori*, but *H. pylori* was absent following eradication treatment or spontaneous loss due to advanced atrophy).

Lesion morphologies and depth of invasion were classified according to the Japanese Classification of Gastric Carcinoma^23,24^. Briefly, polypoid-protruding tumors were classified as type 0–I. Slightly elevated superficial tumors were defined as type 0–IIa. Superficial flat tumors without elevation or depression were classified as type 0–IIb. Slightly depressed tumors were defined as type 0–IIc. Excavated tumors with depressions were classified as type 0–III. In terms of the invasion depth, T1a represents a tumor confined to the mucosa, T1b1 indicates a tumor with invasion < 500 μm from the muscularis mucosa, and T1b2 is a tumor invading ≥ 500 μm from the muscularis mucosa. The follow-up period was defined as the time from endoscopic or surgical resection of gastric lesions to death from any cause or the last hospital visit. The endoscopic follow-up period was defined as the time from endoscopic or surgical resection of gastric lesions to the last esophagogastroduodenoscopy examination.

The primary purpose of this study was to measure the prevalence of submucosal (T1b1/T1b2), vascular, and lymphatic invasion in oxyntic gland adenoma and GA-FG patients in order to determine the appropriate initial treatment strategy. Patients’ clinical and endoscopic characteristics, as well as their prognostic outcomes, were also reviewed.

This study was approved by the Ethics Committees of Okayama University Hospital and other institutions and adhered to the Declaration of Helsinki. Written informed consent was waived because of the observational, non-interventional, and retrospective design. All investigations were performed in accordance with the relevant guidelines and regulations.

## Results

The patient characteristics are listed in Table 1. This study included 75 men and 41 women. The mean age at diagnosis of oxyntic gland adenoma or GA-FG was 66.4 years (range 46–91 years), and 101 patients were 60 years or older (87.1%). Most patients (n = 108, 93.1%) had a single lesion, six patients (5.2%) had two lesions, and two patients (1.7%) had three lesions. Among the 8 patients with multifocal lesions, synchronous occurrence of oxyntic gland adenoma or GA-FG was observed in 7 patients, while the remaining patients presented with two metachronously developed lesions that were diagnosed 13 months apart. Thus, 126 lesions of oxyntic gland adenoma or GA-FG were included in this study.

**Table 1:** Clinical characteristics of the study population.

	N	%
**Sex**		
Male	75	64.7
Female	41	35.3
Mean age (range), years	66.4 (46–91)	
**No. of lesions**		
1	108	93.1
2	6	5.2
3	2	1.7
***H. pylori infection status***		
Uninfected	38	30.2
Active gastritis	13	10.3
Inactive gastritis	60	47.6
Undeterminable	15	11.9
**Treatments**		
ESD	72	57.1
EMR	42	33.3
Surgery	3	2.4
ESD followed by surgery	2	1.6
None	7	5.6
Median follow-up period (range), months	14.5 (0–107)	
Median endoscopic follow-up period (range), months*	11 (0–107)	
**Outcome**		
Alive	112	96.6
Died of other cause	3	2.6
Unknown	1	0.9

*n = 113. ESD, endoscopic submucosal dissection; *EMR* endoscopic mucosal resection using a diathermic snare.

**Figure 1:**
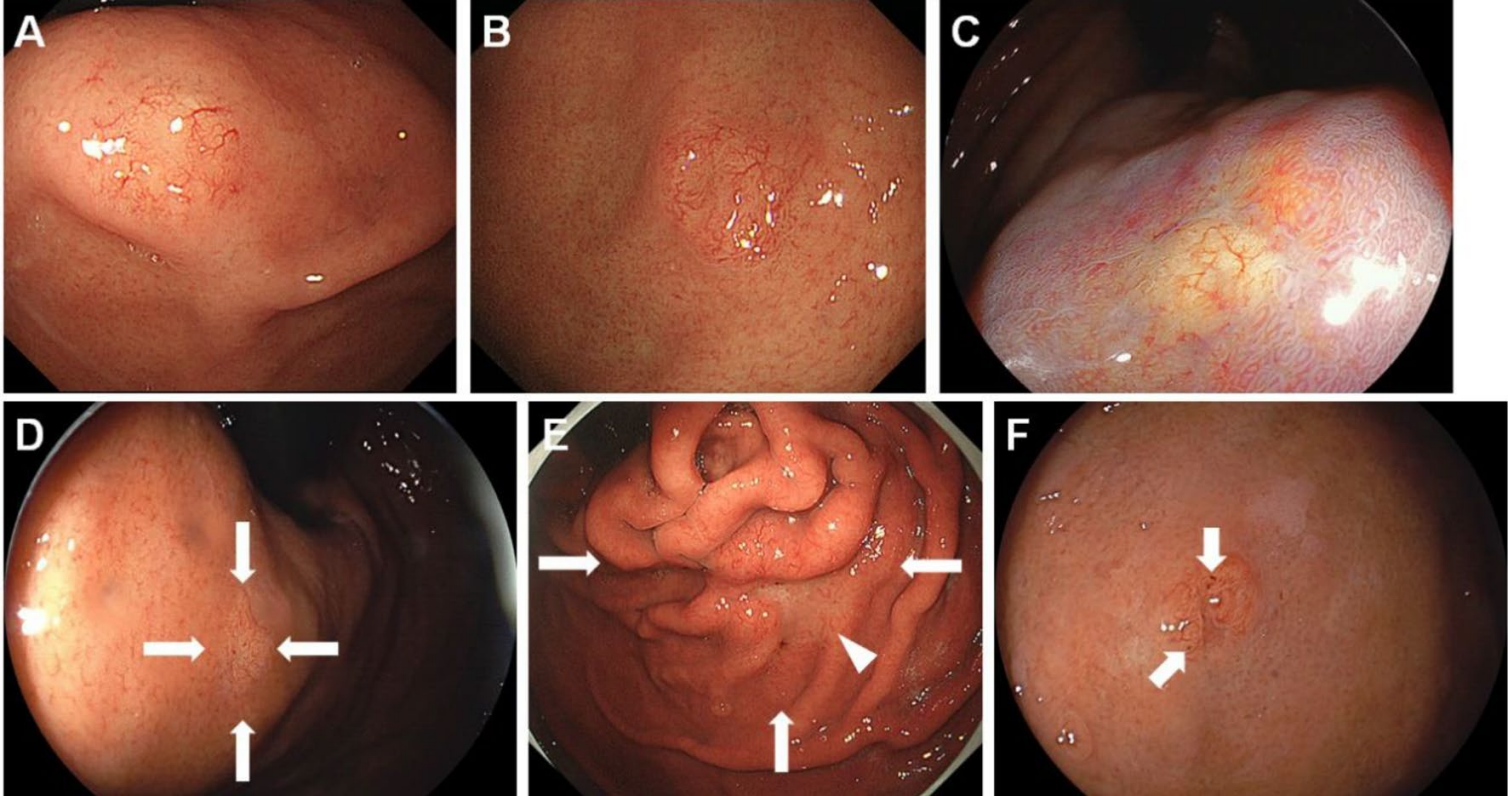
Representative endoscopic images of oxyntic gland adenoma and/or gastric adenocarcinoma of fundic-gland type. (**A**) A typical image of oxyntic gland adenoma showing a slightly whitish, elevated lesion of 5 mm, with vascular dilatation on the surface. (**B**) A representative image of gastric adenocarcinoma of fundic-gland type (T1b1) presented as a slightly yellowish, elevated lesion of 7 mm, with vascular dilatation. (**C**) An oxyntic gland adenoma showing a flat, whitish lesion (linked color imaging). (**D**) A gastric adenocarcinoma of fundic-gland type (pT1b1) showing a flat lesion. The color of the lesion is similar to that of the peripheral gastric mucosa. (**E**) Gastric adenocarcinoma of fundic-gland type (T1b2) presented as the largest tumor (25 mm, arrows). The lesion is composed of swollen gastric folds and a whitish, depressed area in the center (arrowhead). Vascular dilatation is observed throughout the lesion. (**F**) A gastric adenocarcinoma of fundic-gland type (pT1b1) accompanying black pigmentation (arrows).

Representative endoscopic images of oxyntic gland adenoma and GA-FG are shown in Fig. 1, and the endoscopic features of the 126 lesions are listed in Table 2. Oxyntic gland adenoma and GA-FG were most frequently observed in the gastric body (n = 66, 52.4%), followed by the fornix (n = 38, 30.2%) and cardia (n = 21, 16.7%). Among the lesions developing in the gastric body, 34 lesions were found in the upper third portion, 24 lesions in the middle third portion, and 8 lesions in the lower third portion. No lesions were identified in the gastric antrum or pylorus. The mean lesion size was 6.2 mm (range 2–25 mm). Notably, the lesion size was 10 mm or less in 117 lesions (92.9%). Oxyntic gland adenoma and GA-FG appeared as 0–IIa (n = 72, 57.1%), 0–IIb (n = 39, 31.0%), 0–IIc (n = 9, 7.1%), 0–IIa + IIc (n = 4, 3.2%), or 0–I (n = 2, 1.6%). Macroscopically, 64 lesions (50.8%) exhibited a subepithelial lesion. The color of the lesion was similar to that of the peripheral mucosa (n = 49, 38.9%), yellowish-white (n = 34, 27.0%), whitish (n = 22, 17.5%), reddish (n = 14, 11.1%), or yellowish (n = 7, 5.6%). Vascular dilatation was present on the surface of 81 lesions (64.3%), and black pigmentation was observed in 18 lesions (14.3%).

**Table 2:** Endoscopic characteristics of the study population.

	N	%
**Location**		
Fornix	38	30.2
Cardia	21	16.7
Body	66	52.4
Upper third of the body	34	
Middle third of the body	24	
Lower third of the body	8	
Angle	1	0.8
Antrum	0	0.0
Pylorus	0	0.0
Mean size (range), mm	6.2 (2–25)	
**Morphology**		
0–I	2	1.6
0–IIa	72	57.1
0–IIa + IIc	4	3.2
0–IIb	39	31.0
0–IIc	9	7.1
0–III	0	0.0
**Macroscopic appearance**		
SEL-like	64	50.8
Non SEL-like	62	49.2
**Color**		
Similar to the peripheral mucosa	49	38.9
Reddish	14	11.1
Whitish	22	17.5
Yellowish-white	34	27.0
Yellowish	7	5.6
**Vascular dilatation on the surface**		
Present	81	64.3
Absent	45	35.7
**Black pigmentation**		
Present	18	14.3
Absent	108	85.7

**Figure 2:**
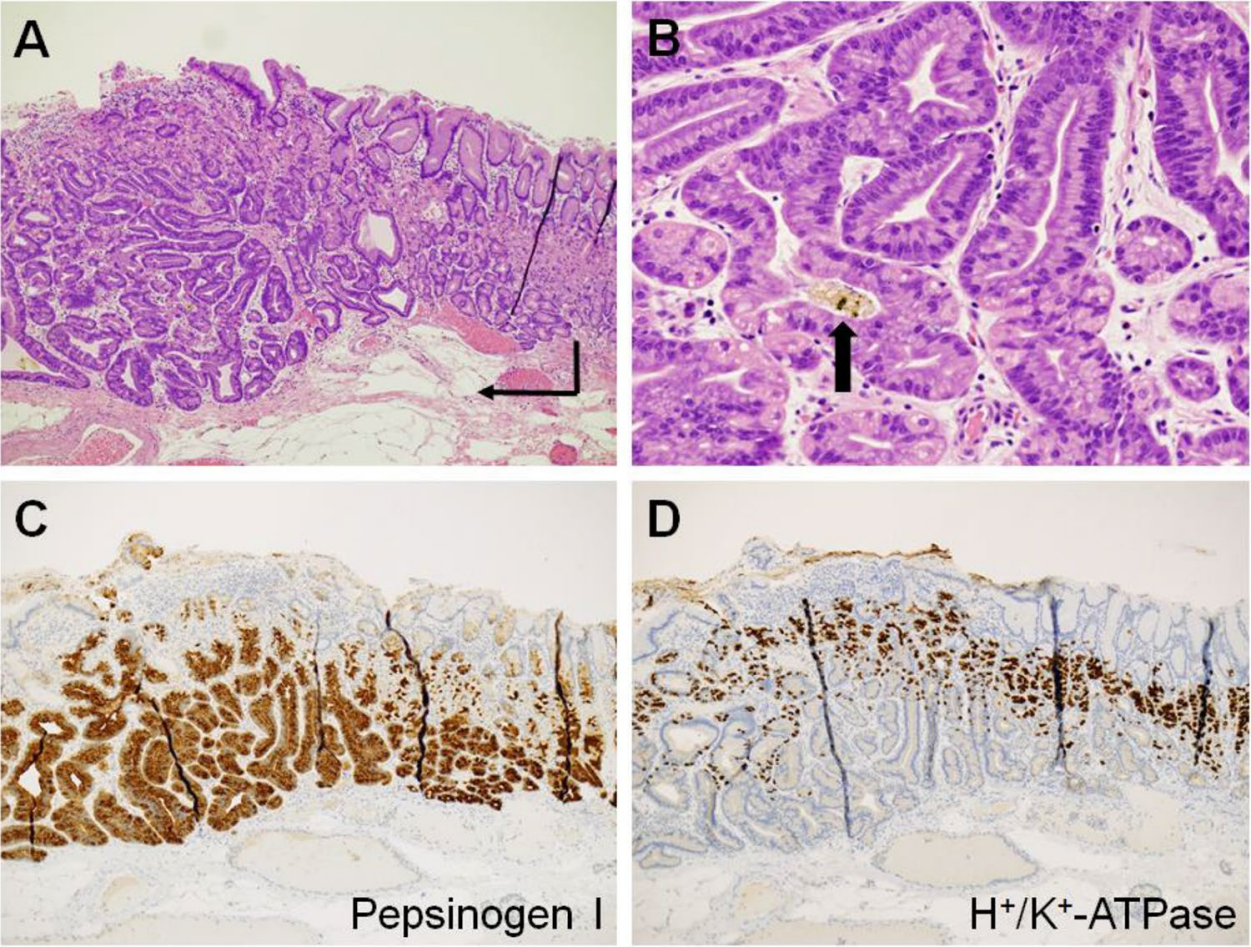
Typical histological images of a gastric adenocarcinoma of fundic-gland type (pT1b1). Endoscopic submucosal dissection specimen of Fig. 1F. (**A**) Neoplastic cells form a more complex gland structure (hematoxylin and eosin staining, × 4.2, arrow), compared to their normal oxyntic counterparts (right portion of image). The neoplastic cells are mainly observed in the deeper portion of the mucosa and covered with foveolar epithelium. (**B**) Tumor cells are composed of highly differentiated columnar cells with mild nuclear atypia and a pale basophilic cytoplasm (hematoxylin and eosin staining, × 20). Brown to black pigments are observed in the dilated glands lined by the neoplastic cells (arrow). (**C**) The tumor cells are positive for pepsinogen-I. (**D**) The tumor cells are partially positive for H^+^/K^+^-ATPase.

The histological features of oxyntic gland adenoma and GA-FG are listed in Table 3, and typical histological images of GA-FG are shown in Fig. 2. Information on the depth of invasion was available for 107 lesions; 51 lesions were T1a (40.5%), 48 lesions were T1b1 (38.1%), and 8 lesions were T1b2 (6.3%). No lesions invaded the muscularis propria or deeper. Vascular invasion was present in 1/104 lesions (0.8%), and lymphatic invasion was present in 1/106 lesions (0.8%). Immunohistochemistry showed that 67/71 lesions were positive for pepsinogen I (94.4%), 71/82 lesions were positive for MUC6 (86.6%), of 24/39 lesions were positive for H^+^/K^+^-ATPase (61.5%), and 9/73 lesions were positive for MUC5AC (12.3%). No lesions were positive for MUC2 (0/23, 0.0%) or CD10 (0/15, 0.0%).

**Table 3:** Histological characteristics of the study population.

	N	%
**Depth of invasion**		
T1a	51	40.5
T1b1	48	38.1
T1b2	8	6.3
Not available	19	15.1
**Vascular invasion**		
Present	1	0.8
Absent	103	81.7
Not available	22	17.5
**Lymphatic invasion**		
Present	1	0.8
Absent	105	83.3
Not available	20	15.9
**Pepsinogen I**		
Positive	67	94.4
Negative	4	5.6
**MUC6**		
Positive	71	86.6
Negative	11	13.4
**H**^**+**^**/K**^**+**^**-ATPase**		
Positive	24	61.5
Negative	15	38.5
**MUC5AC**		
Positive	9	12.3
Negative	64	87.7
**MUC2**		
Positive	0	0.0
Negative	23	100.0
**CD10**		
Positive	0	0.0
Negative	15	100.0

Oxyntic gland adenoma and GA-FG were resected by endoscopic submucosal dissection (ESD, n = 72, 57.1%), endoscopic mucosal resection using a diathermic snare (EMR, n = 42, 33.3%), surgery (n = 3, 2.4%), or ESD followed by surgery (n = 2, 1.6%) (Table 1). EMR in one patient resulted in a partially positive horizontal margin, while none of the patients had positive vertical margins. One patient with T1b2 GA-FG underwent additional surgery after ESD according to the attending physician’s recommendation. The other patient with T1b1 GA-FG underwent surgical resection after ESD at the patient’s request. No lymph node metastases were observed in the surgically resected specimens. During the median follow-up period of 14.5 months (range 0–107 months), 112 patients were still alive (96.6%), while three patients died of causes other than oxyntic gland adenoma or GA-FG (2.6%), including cecal cancer, small intestine cancer, and recurrence after cholangiocellular carcinoma. The outcomes of the remaining patients were unavailable. Repeat esophagogastroduodenoscopy was performed in 113 lesions (89.7%), with a median endoscopic follow-up period of 11 months (range 0–107 months). Local recurrence was identified in 2 patients. Although the initial lesion was confined to the mucosa (T1a), EMR in one patient resulted in a positive horizontal margin, and recurrence of oxyntic gland adenoma was noted on the stomach scar 45 months after the initial treatment. The other patient also had recurrence of oxyntic gland adenoma on the stomach scar, despite curative resection of the initial lesion by ESD with negative horizontal and vertical margins and no lymphatic or vascular invasion. Both patients underwent no additional treatment and were followed up with endoscopic and radiological examinations.

When considering the 8 patients with T1b2 GA-FG (Table 4), one patient underwent surgical resection and another patient underwent ESD followed by surgery, while 5 patients were treated with ESD alone, and the remaining patient was treated with EMR alone. In the 6 patients who underwent ESD or EMR alone, no distant metastases or recurrences were identified during the median endoscopic follow-up period of 10.5 months (0–54 months).

**Table 4:** Characteristics of the patients with submucosal invasion of 500 μm or more (T1b2).

Case no	Sex	Age at diagnosis (years)	Morphology	Size (mm)	Treatment	Submucosal invasion (μm)	Endoscopic follow-up period (mo.)	Recurrence	Follow-up period (mo.)	Outcome
1	F	76	0–IIa	7	ESD	870	29	None	61	Alive
2	F	66	0–IIa + IIc	15	ESD	2700	54	None	64	Dead by other cause
3	M	59	0–IIa + IIc	10	ESD	880	19	None	21	Alive
4	M	86	0–IIa	15	ESD	900	0	None	2	Alive
5	M	64	0–IIa	12	ESD	649	0	None	0	Alive
6	M	55	0–I	5	EMR	640	2	None	2	Alive
7	M	46	0–IIa + IIc	25	Surgery	> 500	N/A	None	0	Alive
8	M	61	0–IIa + IIc	15	ESD followed by surgery	> 500	14	None	14	Alive

*F* female, *M* male, *ESD* endoscopic submucosal dissection, *EMR* endoscopic mucosal resection using a diathermic snare, *N/A* not applicable.

## Discussion

To our knowledge, this study included the largest number of patients with oxyntic gland adenoma and/or GA-FG. Based on histological analyses, 51 lesions were confined to the muscularis propria, and submucosal invasion was identified in 56 lesions. Thus, the prevalence of oxyntic gland adenoma and GA-FG was approximately equal in this study. In 111 reported cases, Benedict et al. showed that the invasion depth reached the mucosa (n = 31), muscularis mucosae (n = 1), submucosa (n = 63), serosa (n = 1), muscularis propria (n = 1), and undetermined (n = 14)^25^. Thus, the incidence rates of oxyntic gland adenoma and GA-FG were estimated to be 1:2^22^. The discrepancy in the prevalence between our study and previous reports may have been caused by publication biases because more lesions with submucosal invasion (GA-FG) may be published than those confined within the mucosa (oxyntic gland adenoma). In the present study, most of the lesions were resected using either ESD or EMR (n = 114, 90.5%). There were no lesions with invasion at the level of the muscularis propria or deeper, and lymphovascular invasion was present in only 1.6% of lesions. Although EMR resulted in a positive horizontal margin in one patient, there were no lesions with exposed tumor cells in the vertical margins of the endoscopic resection specimens. These results indicate that endoscopic resection is a suitable initial treatment strategy for oxyntic gland adenoma and GA-FG.

Notably, 6 of the 8 GA-FG patients with deep submucosal invasion (T1b2) were followed up without additional surgical resection after endoscopic treatment. No recurrence was documented at a median endoscopic follow-up period of 10.5 months. In Japanese gastric cancer treatment guidelines, endoscopically resected-T1b2 tumors are defined as endoscopic curability C-2 (eCuraC-2), and gastrectomy with lymphadenectomy should be considered^23,24^. Despite the recommendations described in these guidelines, most attending physicians followed up with the patients. These decisions were probably supported by the fact that the neoplastic cells in the submucosa can be regarded as “prolapse-type” misplaced glands showing minimal desmoplastic changes, rather than true submucosal invasion^21,25^. However, further investigations with longer observation periods are required to elucidate the appropriate treatment strategies for oxyntic gland adenoma and GA-FG.

Although some debate remains regarding the biological behavior of oxyntic gland adenoma and GA-FG, it is widely accepted that this gastric neoplasm rarely affects lymphatic and vascular systems and grows slowly^8,9,12,14,15^. Lymphatic and/or vascular invasion was positive in 7/94 reported cases (7.4%)^25^ and 2/126 in the present study (1.6%). Additionally, no patients died of oxyntic gland adenoma or GA-FG in our study or in previous reports. These data suggest that oxyntic gland adenoma and GA-FG are neoplasms with low malignant potential, resulting in a favorable prognosis. Thus, discussions are still ongoing on the correct nomenclature^25^ and some pathologists favor terms, such as “oxyntic gland adenomas,” “fundic gland adenoma,” or “fundic gland polyps with chief or parietal cell differentiation,” in place of “adenocarcinoma.” In contrast, a patient with GA-FG infiltrating into the subserosa with lymphatic and venous invasion has been reported^10^. Ushiku et al. proposed that GA-FG with atypical cellular differentiation, such as mucous neck cells and foveolar epithelium in addition to chief cells and parietal cells, be subcategorized as “adenocarcinoma of fundic gland mucosa type” as they are probably a more aggressive phenotype^21^. Since lesions of oxyntic gland adenoma and GA-FG are likely to be heterogeneous, it is necessary to establish appropriate histological subcategorization and terminology.

We revealed that these neoplasms developed in patients aged 60 years or older in most cases (87.1%), with a male-to-female ratio of approximately 2:1. Male predominance and frequent occurrence in patients aged 60 years or older has been previously reported^14,22,25^. Oxyntic gland adenoma and GA-FG mostly occurred solely (93.1%), with the development of two or three lesions occurring in a limited number of patients. As the second and third lesions were identified within 12 months from the diagnosis of the initial lesion, except in one patient, careful observation is recommended during esophagogastroduodenoscopy to avoid overlooking other lesions of oxyntic gland adenoma and GA-FG. *H. pylori* infection reportedly has no association with the development of these lesions^22,25^. Chiba et al. reported that among 20 patients with oxyntic gland adenoma or GA-FG, 5 patients were uninfected with *H. pylori* (25.0%), while the remaining 15 patients had active or inactive gastritis (75.0%)^14^. We also revealed that the *H. pylori infection* status varied from inactive gastritis (47.6%) and uninfected (30.2%) to active gastritis (10.3%). These results reinforce the hypothesis that oxyntic gland adenoma or GA-FG develops irrespective of *H. pylori* infection status.

The typical endoscopic features of oxyntic gland adenoma or GA-FG are known as a whitish small elevation of < 10 mm in diameter, with irregular branching vessels, predominantly occurring in the upper third portion of the stomach^14,22,25^. In the present study, 73.8% of the lesions were found in the upper third portion of the stomach (i.e., the fornix, cardia, and upper third of the body), while the other lesions were identified in the middle third portion (i.e., the middle to lower third of the body and angle). No lesions were observed in the lower third portion of the stomach. The lesion size was 10 mm or less in most cases (92.9%). A slightly elevated superficial morphology accounted for more than half of the lesions (57.1%), and a flat tumor, without elevation or depression, represented approximately one-third of the lesions (31.0%). Half of the lesions showed a subepithelial lesion (50.8%). Vascular dilatation was present on the surface of approximately two-thirds of the lesions (64.3%). Although the color of the lesions in oxyntic gland adenoma and GA-FG varied from a color similar to the peripheral mucosa, to yellowish-white, white, reddish, and yellowish, the macroscopic features observed in the present study were consistent with those reported previously^22,25^. Brownish or black pigmentation has been described in several case reports^12,17,19^, which are considered to reflect pigmented substances contained in the dilated glands of neoplastic cells (Fig. 2B). Such pigmentation was identified in 14.3% of lesions in this study.

This study had several limitations. First, endoscopic and histological analyses were performed at various institutions. It is possible that interobserver variations and differences in methodologies between the participating institutions may have resulted in heterogeneous patient data. However, our results could be generalized to institutions worldwide. Second, the follow-up period following diagnosis of oxyntic gland adenoma and/or GA-FG was relatively short (median 14.5 months), resulting in insufficient analysis of patient outcomes. Complete follow-up with a longer observation period should be considered in future investigations. Third, immunohistochemical studies were not performed for all patients. Although diagnosis can essentially be made according to the WHO classification criteria based on the presence of intramucosal proliferation of differentiated columnar cells with pale basophilic cytoplasm and mild nuclear atypia that mimics the oxyntic gland, it is preferably, however, confirmed by positivity for both pepsinogen I and MUC6^22^. Cell differentiation markers, such as H^+^/K^+^-ATPase (parietal cell) and MUC5AC (foveolar epithelium), will help in the diagnosis of the adenocarcinoma of the fundic gland mucosa subtype^21^. In addition, D2-40-based assessment improves the histopathological detection of lymphovascular invasion. Fourth, histological and patient information was incomplete due to the retrospective nature of this study; the mucosa adjacent to oxyntic gland adenoma or GA-FG is reportedly normal without any intestinal metaplasia or atrophy^9^. Additionally, several researchers have suggested a possible relationship between acid suppressive therapy and the pathogenesis of oxyntic gland adenoma and GA-FG. Future studies incorporating such histological and patient information may reveal a more detailed pathophysiology of these gastric neoplasms.

In conclusion, we retrospectively investigated 116 patients with 126 lesions of oxyntic gland adenoma or GA-FG. The majority of oxyntic gland adenomas and GA-FG cases were resected using ESD or EMR. Histologically, there were no lesions with invasion at the level of the muscularis propria or deeper, and lymphovascular invasion was present in only 1.6% of lesions. Of the ESD/EMR specimens, no lesions were positive for vertical margins. Based on these results, we believe that endoscopic resection is a suitable initial treatment strategy for oxyntic gland adenoma and GA-FG. Although most of the T1b2 GA-FG patients were treated with endoscopic resection alone and no recurrence was documented, further investigations are required to elucidate the optimal treatment strategy for T1b2 GA-FG.

## Data Availability

The datasets generated and/or analyzed during the current study are available from the corresponding author upon reasonable request.

## Abbreviations

eCuraC-2: Endoscopic curability C-2
EMR: Endoscopic mucosal resection using a diathermic snare
ESD: Endoscopic submucosal dissection
GA-FG: Gastric adenocarcinoma of the fundic gland type
